# Epitypification of the Central African *Cantharellusdensifolius* and *C.luteopunctatus* allows for the recognition of two additional species

**DOI:** 10.3897/mycokeys.49.32034

**Published:** 2019-03-27

**Authors:** Bart Buyck, Terry W. Henkel, Valérie Hofstetter

**Affiliations:** 1 Institut de Systematique, Evolution, Biodiversite (ISYEB – UMR 7205), Museum national d’Histoire naturelle, Sorbonne Université, CNRS, CP 39, 12 Rue Buffon, F-75005 Paris, France Sorbonne Université Paris France; 2 Department of Biological Sciences, Humboldt State University, Arcata, California, 95521, USA Humboldt State University Arcata United States of America; 3 Department of Plant Protection, Agroscope Changins-Wädenswil Research Station ACW, Rte De Duiller, CH-1260 Nyon 1, Switzerland Wädenswil Research Station Nyon Switzerland

**Keywords:** Cantharellales, ectomycorrhizal, *tef*-1, phylogeny, rain forest, taxonomy

## Abstract

*Cantharellusdensifolius* and *C.luteopunctatus* are epitypified on the basis of recently collected specimens from the Central African rain forest that correspond in every way to their respective original descriptions. Sequences obtained from these new collections demonstrate that both epitypes represent distinct species that belong in different subclades of Cantharellussubg.Rubrinus. Previously, the name *C.densifolius* has been consistently misapplied to more or less similar species from the African woodland area, including *C.densilamellatus***sp. nov.** which is described here, In addition, *C.tomentosoides***sp. nov.**, a rain forest species that is easily confused with *C.densifolius*, is described.

## Introduction

Tropical African *Cantharellus* species (“chanterelles”) have been well-documented compared to other tropical regions. Nonetheless, there is a great need for sequence data to provide the foundation for unambiguous species concepts. This is due to the highly variable and potentially deceptive macromorphologies, compounded by the limited interspecific micromorphological variation among *Cantharellus* species (Buyck et al. 2014, 2016e, Olariaga et al. 2015).

Despite this need for sequence data, *Cantharellus* has been difficult to work with from a molecular standpoint. *Cantharellus* ribosomal genes have high rates of molecular evolution (Moncalvo et al. 2006) and *Cantharellus* species often have an unusually long ITS sequence, ranging from around 900 to 2200 base pairs. The ITS barcode locus is consequently difficult to obtain for chanterelles (Schoch et al. 2012). This is especially true for old type specimens, due to their degraded DNA and resulting difficulties in extraction, and the frequent failures in the annealing of fungal primers designed to amplify the ITS locus or part of it.

While phylogenetic understanding of *Cantharellus* in Europe and North America has recently improved (Buyck et al. 2016c,d,e,f; Olariaga et al. 2015, 2016), the continuing lack of sequence data for Old World *Cantharellus* has helped to perpetuate taxonomic confusion regarding species delimitation and infrageneric relationships (Buyck et al. 2013, 2014). For Africa, some *Cantharellus* species from Madagascar and the African mainland have been circumscribed by single or multilocus molecular phylogenies (Ariyawansa et al. 2015, Buyck et al. 2014, 2015, 2016a, 2017, Liu et al. 2015, De Kesel et al. 2016). However, species recognition for the majority of chanterelles from the Guineo-Congolian rain forest is still based on morphological descriptions published over half a century ago (Heinemann 1958, 1959, 1966).

Many of the older species names for African chanterelles have been misapplied to morphologically similar specimens gathered in dense, closed-canopy rain forest versus the surrounding seasonal woodlands, or in open woodlands of neighboring Madagascar (Buyck et al. 2016g). As type specimen DNA of these earliest described rain forest chanterelles appears completely degraded (fide De Kesel et al. 2016), epitypification with sequencing of newly collected specimens is the most efficient way for unambiguous species delimitation. Until recently, new, well-documented specimens of rain forest chanterelles have not been available for sequencing. Thanks to renewed collecting efforts for *Cantharellus* in the African rain forest, the limits of species bearing these older names can be assessed, and the epitypification process has begun (Buyck et al. 2016a,b,g, De Kesel et al. 2016, Buyck and Hofstetter 2018).

Here we epitypify *Cantharellusdensifolius* Heinem. based on recent collections made ~400 km from the type locality but in the same forest habitat. The chosen epitype, which is in perfect agreement with the original description, clearly demonstrates that the name has been misapplied to different species for decades. The new collections constitute the first records for *C.densifolius* since this species was collected by Mme. Goossens-Fontana in 1929 and later described by Heinemann (1958). In this paper we also epitypify *Cantharellusluteopunctatus* Heinem, previously considered a yellowish color-variant of *C.densifolius* (Eyssartier 2001), but shown here to be a morphologically well-defined, independent species. Additionally, two new species previously confused with *C.densifolius* are described.

## Material and methods

### Collecting and macromorphology

Basidiomata were collected in the Central African Republic (RCA) during dry conditions in early May 2016 in pure *Gilbertiodendrondewevrei* stands of the Dzangha-Sangha Forest Reserve. In Cameroon, basidiomata were collected during the Aug.-Nov. rainy seasons of 2014, 2016, and 2017 from the Dja Biosphere Reserve, Northwest Sector near the village of Somalomo, Upper Dja River Basin, within a two km radius of a base camp located at 3°21'29.8"N, 12°43'46.9"E, 650 m a.s.l., in forests dominated by *G.dewevrei*. Photographs and descriptions of macromorphological features were made from fresh material in the field. Colors were compared with color plates from Kornerup and Wanscher (1978) and are cited in parentheses. Collections were dried with a self-made drier (RCA) or silica gel (Cameroon). Epitype material and additional specimens are deposited in PC, Museum national d’histoire naturelle, Paris, and for the Cameroon collections also in the following herbaria: YA, Cameroon National Herbarium; HSC, Humboldt State University.

### Micromorphology

Microscopic observations and measurements were made from ammoniacal Congo red mounts after a short pretreatment in a 10% aqueous KOH solution to improve tissue dissociation and matrix dissolution. Original drawings for all elements of the hymenium and pileipellis were made at a magnification of 2400× with the aid of a camera lucida. Measurements of basidiospores cite length, width and length/width ratio (Q) in this format: (minimum–) mean minus standard deviation – mean value – mean plus standard deviation (− maximum measured); basidiospore size statistics are based on 20 basidiospores measured per specimen.

### Taxon sampling and phylogenetic analyses

Genomic DNA isolation, amplification and sequencing for the transcription elongation factor 1-alpha (*tef*-1) of the new *Cantharellus* collections were obtained as described in Buyck et al. (2014). The *tef*-1 sequence data from other taxa were obtained from our previous publications (Buyck et al. 2014, 2016a, b, 2018; Das et al. 2018). Sequences were assembled and corrected with the software package Sequencher 3.0 (Gene Codes Corp., USA). Alignment of *tef*-1 was performed manually in MacClade 4.05 (Maddison and Maddison 2002). Searches for the optimal tree and branch robustness were conducted with the program PhyML (Guindon and Gascuel 2003), under a GTR nucleotide substitution model, with the search starting from a distance-based tree and with the proportion of invariable sites, gamma shape parameter and number of substitution categories estimated during the search. Three independent runs were conducted to check for convergence toward the same likelihood value. Branch support was estimated based on 500 bootstrap replicates (ML-bs) and was considered significant when ≥ 70% (Mason-Gamer and Kellogg 1996; Alfaro et al. 2003).

## Results

Seven new sequences were produced for this study (five collections of *C.densifolius*, one of *C.tomentosoides*, and one of *C.luteopunctatus*). The alignment used for phylogenetic analyses included sequences of 90 *Cantharellus* specimens and one of *Craterellustubaeformis* used for outgroup. The full alignment length was 864 base pairs. After exclusion of three spliceosomal introns, the remaining 629 characters were used for the analyses.

**Figure 1.**
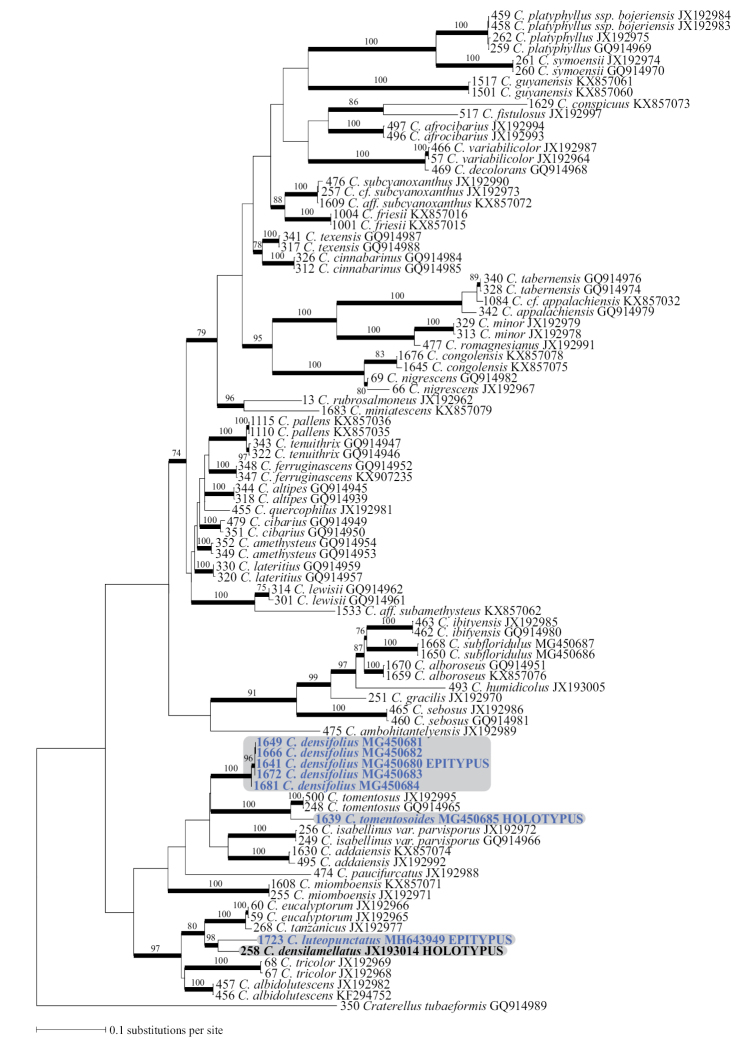
Most likely tree obtained by analysis of the 91 *tef*-1 sequence dataset. Species names are preceded by their extraction number (see Buyck et al. 2014 for corresponding vouchers) and followed by the corresponding GenBank deposit number. Branches that received significant ML bootstrap support are in bold with ML-bs associated values indicated above the branches. Newly produced sequences are in blue and discussed species are in bold.

The most likely tree (Fig. 1; -ln = 14270.90232) placed *C.densifolius* (ML-bs = 100 %) as sister without support to a highly supported monophyletic group (ML-bs = 100 %) containing *C.tomentosoides* sp. nov. and the typical woodland species *C.tomentosus* (ML-bs = 100 %) (Buyck et al. 2000). *Cantharellusdensilamellatus* sp. nov. was not conspecific with *C.densifolius*, instead forming a highly supported terminal clade (ML-bs = 98 %) with the designated epitype of *C.luteopunctatus*, and this clade was supported (ML-bs = 80 %) as sister to a clade containing *C.tanzanicus* and the Malagasy, eucalypt-associated *C.eucalyptorum* Buyck & V. Hofst. (Ariyawansa et al. 2015). These species formed a larger, strongly supported clade (ML-bs = 97 %) within subg. Rubrinus with two additional Malagasy species, the woodland endemic *C.albidolutescens* Buyck & V. Hofst. (Buyck et al. 2015), and the eucalypt-associated *C.tricolor* Buyck & V. Hofst. (Ariyawansa et al. 2015).

### Taxonomy

#### Cantharellus densifolius

Taxon classificationFungiScleractiniaFungiidae

Heinem., Bull. Jard. bot. État Brux. 28(4): 410. 1958.

Figs 2 , 3

##### Original diagnosis.

“*Pileus carnosulus, infundibuliformis, lobatus, laete ochraceus, squamulosus. Stipes solidus, pileo concolor. Lamellae confertissimae, angustissimae, furcatae, non intervenatae. Caro ochracea, sapore amaro. Sporae breviter ellipsoidae, 5,6–7 × 3,7–4,5 μm.*”

##### Holotype.

DEMOCRATIC REPUBLIC OF THE CONGO. Binga, dispersed on the soil of the dry forest, Aug. 1929, Mme. Goossens-Fontana 879 (BR).

##### Iconography.

Heinemann (1958, fig. 45; 1959, pl. XXVI, fig. 11).

##### Original description.

(freely translated from French) “Pileus ca. 8 cm diam., thin, deeply concave to infundibuliform, with the margin convex to stretched, irregular and wavy; surface ochraceous orange, very finely punctuated with tiny squamules that are easily detached. Stipe ca. 30 × 7 mm, cylindrical, massive, concolorous with the cap. Hymenophore composed of crowded gill-folds, less than 1 mm high, 1–4 times forking, deeply decurrent, with blunt gill edge, not interveined. Context fibrous, bright ochraceous. Taste bitter. Smell of *C.cibarius*. Spore print white. Exsiccatum with reddish ochre brown color.

Spores hyaline, 5.6–7 × 3.7–4.5 μm, shortly ellipsoid, thin-walled, not amyloid; apiculus small. Basidia slender, 37–48 × 6–8 μm, probably 6-spored. Hymenophoral trama pseudoparenchymatic, slightly bilateral. Pseudoparenchyma very compact. Pileipellis squamulae composed of easily detaching cells that are irregularly cylindrical, often undulating, thick-walled with a very thick yellow wall in ammonium solution; the terminal cells obtusely rounded. Clamp connections rare.”

**Figure 2.**
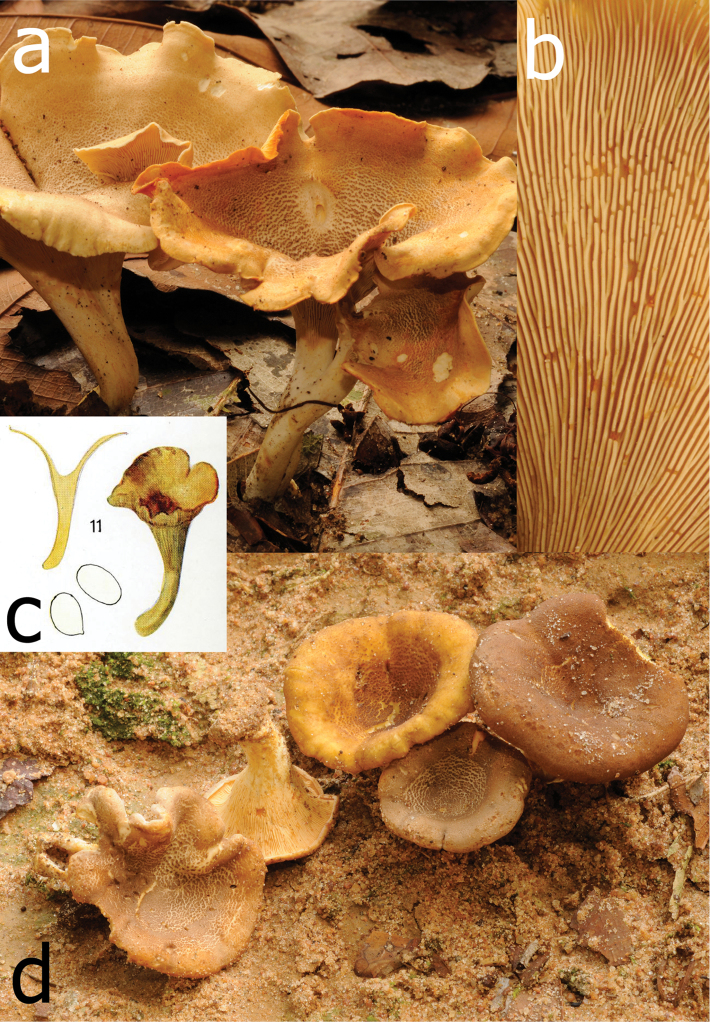
*Cantharellusdensifolius*. **a** Field habit of the epitype (BB 16.021), showing the areolate-squamose ochraceous orange pileus surface resulting from the concentrical disruption of a dark tomentum that covered initially the young pileus **b** Detail of the epitype hymenophore showing the remarkably low and thick, crowded, repeatedly forking gill folds without interstitial veination **c** Original watercolor of the holotype by Mme. Goossens-Fontana from Heinemann (1959), reproduced with the permission of Botanic Garden Meise, Belgium **d** Field habit of specimen BB 16.081 showing the variability of the pileus color within a single collection. Photos: B. Buyck.

**Figure 3.**
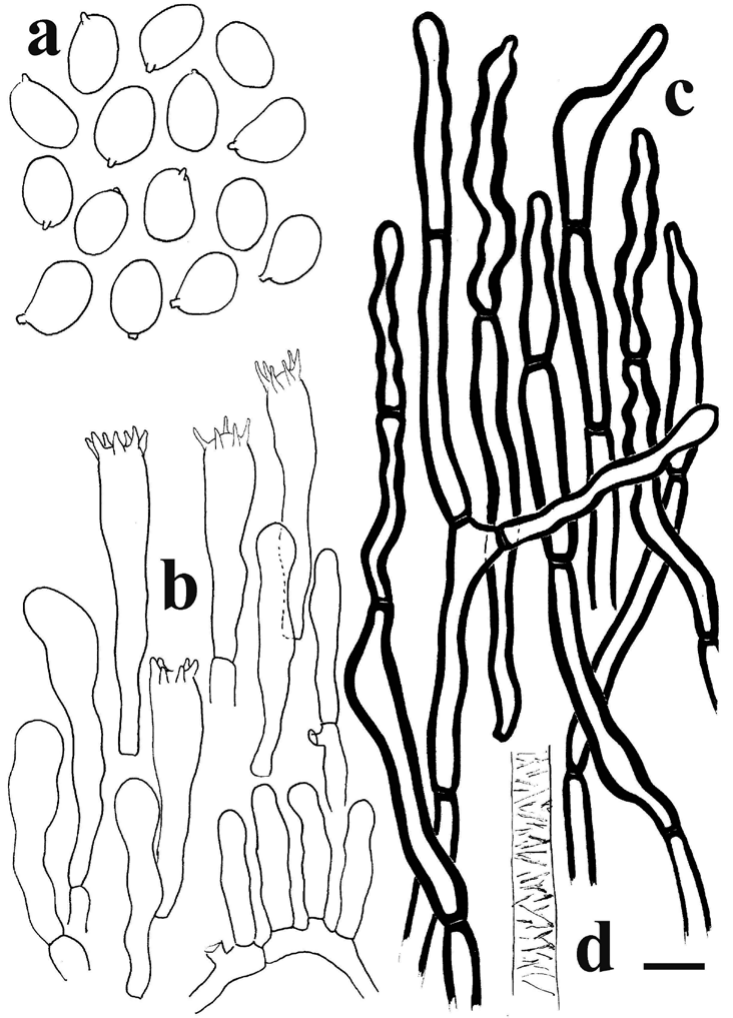
*Cantharellusdensifolius* (epitype, BB 16.021). Microscopic features: **a** basidiospores **b** basidia and basidiola **c** distinctly thick-walled and typically sinuous-undulate hyphal extremities of the pileipellis **d** detail of an encrusted hypha from the pileus context. Scale bar: 10 µm but only 5 µm for basidiospores. Drawings B. Buyck.

##### Description of the epitype.

*Basidiomata* solitary or in small groups. *Pileus* medium-sized to rather large and up to 100 mm diam., 1–2 mm thick at mid-radius, yet firm and leathery; margin undulating, irregularly waving to strongly lobed, smooth; surface layer remaining more or less continuous in the center, then disrupting toward the margin with expansion of the pileus and forming dark, more or less concentrically arranged squamules or fibres; observed under a hand lens these can be appressed and flat, or forming a woolly-cottony mass of suberect fibers, pale brown (5AB3) to warm chocolate brown or dark brown (5EF7–8, 5F4–8, 5D5–8, 5C5–6) when young, but rapidly tinged with ochraceous orange as a consequence of the exposure of the underlying pileus tissue and the yellowing tendency of the context. *Hymenophore* composed of very crowded (>30/mm) gill folds, which are very low (<1 mm) and thick, not interveined, often transversely fissuring over their entire height, repeatedly forking, strongly decurrent, off-white when young, then darkening to the color of coffee with copious milk, moderately to strongly yellowing upon handling. *Stipe* 40–60 × 4–5 mm, widening toward the hymenophore and there up to 8(–17) mm wide; surface smooth, whitish, pale brown just beneath the hymenophore. *Context* whitish, thin and leathery, fibrous in the stipe, faintly to strongly yellowing upon injury or handling, occasionally turning rusty brown. *Odor* faint. *Taste* mild. *Spore print* off-white.

*Basidiospores* ellipsoid, (5.8–)6.0–6.46–6.9(–7.1) × (3.5–)3.8–4.19–4.6(–5.0) µm, Q = (1.3–)1.4–1.55–1.7(–1.8), smooth, hyaline. *Basidia* mostly 35–50 × 7–8 µm, (5–)6(–7)-spored; sterigmata stout but rather short. *Subhymenium* forming a very dense layer, not pseudoparenchymatous, but composed of mostly short cells that are not wider than the basidium base. *Cystidia* none. *Pileipellis* of loosely interwoven and much septate hyphal extremities composed of ramifying chains of distinctly thick-walled cells; terminal (but also sometimes subapical) cells subcylindrical, but often more irregularly inflated or sinuous-tortuous in outline, 5–8(–10) μm wide, mostly 25–45 µm long, often narrowing or abruptly constricted near the apex. *Clamp connections* absent.

CENTRAL AFRICAN REPUBLIC. Dzanga-Sangha Forest Reserve, near Bayanga, close to Bai-Hakou base camp, 02.859934N, 16.467492E, in monospecific *Gilbertiodendrondewevrei* forest, on bare sandy soil, 15 May 2016, 1641/Buyck 16.021 (PC 0142486, **epitypus hic designatus**). MycoBank MBT 384669.

##### Additional specimens examined.

CENTRAL AFRICAN REPUBLIC. Dzanga-Sangha Forest Reserve, near Bayanga, in and around Bai-Hakou base camp, 02.859934N, 16.467492E, in monospecific *Gilbertiodendrondewevrei* forest, on bare sandy soil, 19 May 2016, Buyck 16.081/1656 (PC0142487), Buyck 16.065/1649 (PC0142488); ibid., 24 May 2016, Buyck 16.113/1672 (PC0142490); ibid., 26 May 2016, Buyck 16.137/1681 (PC0142489).

##### Discussion.

*Cantharellusdensifolius* was originally described by Heinemann (1958) and was one of three rain forest *Cantharellus* species characterized by crowded gill folds. The other two chanterelles with equally crowded gill folds were the fragile, smaller (pileus < 30 mm diam.), bright orange *C.pseudofriesii* Heinem. and the medium-sized, bright yellow *C.luteopunctatus* Heinem. Eyssartier (2001) re-examined the holotype of each of these three species, and concluded that *C.pseudofriesii* was distinctive due to its possession of clamp connections (contrary to the original description, see also Buyck et al. 2016a), and suggested that *C.luteopunctatus* may be a color variant of *C.densifolius* because of its similar micromorphological features.

The epitype specimen selected here perfectly agrees with the original description of *C.densifolius*. Indeed, Heinemann (l.c.) described it as a medium-sized species with an infundibuliform, ochre-orange and finely squamulose pileus measuring ca. 80 mm diam. and ending in an irregular but stretched margin, with strongly decurrent, crowded, frequently forking and very low gill folds (< 1 mm high) with blunt edges, lacking any intervenation. Heinemann cited shortly ellipsoid basidiospores of near identical size, more precisely given by Eyssartier (2001), as important microscopic features, along with the pileipellis composed of easily disintegrating, very thick-walled hyphal extremities that are sinuous-undulating in outline (compare Heinemann 1958 fig. 45B with our Fig. 3c).

The typical features appear to be quite constant across all specimens of *C.densifolius* examined here, including both the size and shape of basidiospores (Table 1), as well as the undulate, thick-walled, often apically tapered or constricted hyphal extremities (although less so in Buyck 16.137). The ochre-orange color of the pileus described for the holotype was also present in the epitype (Fig. 2a, c) but across collections examined here pileus color ranged from ochraceous yellow over orange to pale brown and even dark chocolate brown, but never to bright lemon yellow as described for *C.luteopunctatus*. This is consistent with the highly variable color within many other *Cantharellus* species (Olariaga et al. 2015, Buyck et al. 2016e). For *C.densifolius*, the general color of the pileus also depends on the degree of yellowing of the context underneath the disrupted surface tomentum, which can vary between or within individual basidiomata.

The form of the hyphal extremities composing the pileal tomentum is very similar to that of various other squamulose species in subg. Rubrinussect.Isabellinus Eyssart. & Buyck, in particular those of the African *C.tanzanicus* Buyck & V. Hofst. (Buyck et al. 2013) and the Malagasy *C.eucalyptorum* Buyck & V. Hofst. and *C.tricolor* Buyck & V. Hofst., the latter two species being associates of introduced eucalypts (Liu et al. 2015). The differences in habitat and basidiospore size allow differentiation of *C.densifolius* from these species.

*Cantharellusdensifolius* has repeatedly been reported from the surrounding woodland area in Africa (e.g. Heinemann 1966, Buyck 1994, Buyck et al. 2000, Härkönen et al. 2015). Our sequence data have now indicated that such woodland specimens merit recognition as independent species. For example, the morphologically similar *C.densilamellatus* sp. nov. described below is unrelated to *C.densifolius* but resolved as sister to *C.luteopunctatus* (Fig. 1).

**Table 1.** Comparison of basidiospore measurements for the discussed species.

* C. densifolius *			
Holotype (Heinemann 1958):	5.6–7	3.7–4.5 µm	
Holotype (Eyssartier 2001):	5.5–**6.37**–7	3.5–**4.06**–4.5	1.3–**1.57**–1.8
Epitype:	(5.8–)6.0–**6.46**–6.9(–7.1)	(3.5–)3.8–**4.19**–4.6(–5.0)	(1.3–)1.4–**1.55**–1.7(–1.8)
Buyck 16.137	(5.4–)5.5–**5.78**–6.1(–6.5)	(3.5–)3.9–**4.14**–1.5(–1.6)	(1.2–)1.3–**1.40**–1.5(–1.6)
Buyck 16.081	(4.8–)5.4–**5.78**–6.1(–6.2)	(3.5–)3.8–**4.02**–1.5(–1.7)	(1.2–)1.3–**1.44**–1.5(–1.6)
* C. tomentosoides *			
Holotype	(5.8–)6.0–**6.36**–6.7(–7.1)	(3.9–)4.0–**4.27**–4.5(–5.0)	(1.3–)1.4–**1.49**–1.6(–1.7)
* C. densilamellatus *			
Holotype:	6.7–**7.05**–7.4(7.9)	(3.3)3.4–**3.65**–3.9(4.0)	(1.7)1.8–**1.94**–2.1(2.3)
* C. luteopunctatus *			
Holotype (Heinemann 1958):	4.9–6.0 (7.5)	3.8–4.6 (5) μm	
Holotype (Eyssartier 2001):	5–**5.97**–7	3.5–**4.19**–5	1.2–**1.42**–1.6
Epitype / Henkel 10285	(5.4–)5.7–**6.04**–6.4(–7.1)	(3.9–)4.0–**4.29**–4.6(–5.0)	(1.2–)1.3–**1.41**–1.5(–1.8)
Henkel 10442:	(5.4–)5.4–**5.94**–6.5(–7.3)	(4.1–)4.2–**4.33**–4.5(–4.8)	(1.2–)1.3–**1.37**–1.5

#### Cantharellus luteopunctatus

Taxon classificationFungiScleractiniaFungiidae

(Beeli) Heinem. Bull. Jard. bot. État Brux. 28(4): 415. 1958.

Figs 4 , 5 , 6

##### Basionym.

*Lentinusluteopunctatus* Beeli, Bull. Soc Roy Bot Belge 60: 160. 1928.

##### Original diagnosis.

“*Pileo carnoso-coriaceo, centro depresso, margine incurvato, luteo; furfuraceo brunneo, 3,5–4 cm. lato; stipite cylindrico-solido, glabro, concolori, 3 × 0,5–0,7 cm, lamellis deccurentibus, luteis; sporis ovoideis 5–6 × 3,5–4 μm, carne lutea.*”

##### Holotype.

DEMOCRATIC REPUBLIC OF CONGO. Central forest district, near Djongo-Akula, dispersed on the soil of the dry *Gilbertiodendrondewevrei* forest, Dec. 1925, Mme. Goossens-Fontana 502 (BR).

##### Iconography.

HEINEMANN (1958, fig. 47; 1959, pl. XXVI, fig. 6).

##### Original description.

(freely translated from French) “Pileus rather thick, 49 mm diam., soon depressed, concave with rounded, then straight margin, bright lemon yellow, punctuated – particularly in the center – with minute brownish squamules. Stipe 30 × 6 mm, [30–50 × 5–11 mm], cylindrical, solid inside, yellow, finally rusty, faintly covered from brownish scales. Gills very crowded, deeply decurrent, very narrow, 0.5–11 mm wide (sic!), yellow, irregularly forked, interconnected by rather abundant transversal anastomoses. Context firm, bright yellow, more orangish near the stipe base. Taste strong, bitter. Spore print white. Exsiccatum orangish brown-ochre.

Spores hyaline, shortly ellipsoid, 4.9–6.0 (7.5) × 3.8–4.6 (5) μm, granular inside, thin-walled, not amyloid, with a small apiculus. Basidia narrowly clavate, 30–40 × 6.7–9.5 μm, 4-spored, perhaps sometimes 6-spored. Hymenium not or only slightly accrescent. Subhymenium narrow. Pseudoparenchyma composed of very long and slender elements, mixed with some oleiferous hyphae that do not color in Congo Red. Pileipellis undifferentiated; squamules formed of hyphae united in bundles made up of yellowish to pinkish cells; terminal cells lanceolate or clavate, 6–13 μm diam. Hyphae not amyloid.”

**Figure 4.**
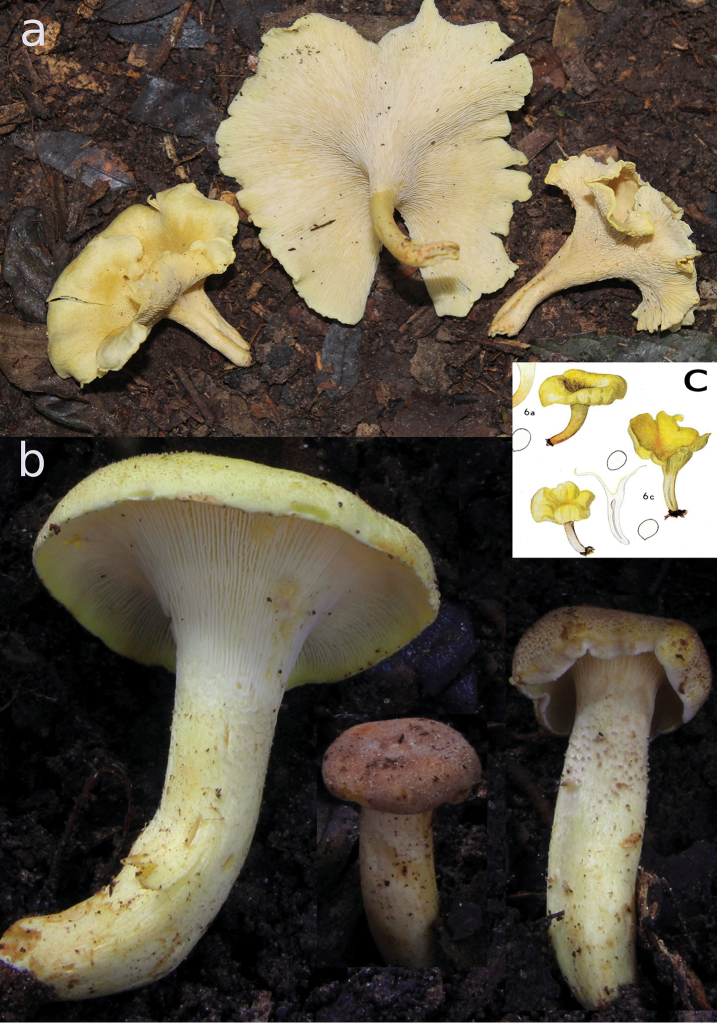
*Cantharellusluteopunctatus*. **a** Field habit of the epitype (TH 10285) **b** details of younger basidiomata from specimen TH 9921, showing the gradual color change of the pileus going from pinkish brown in youngest stages to pale yellow in older stages because of the less dense squamulae; similar squamulae are present on the stipe surface. Composition based on photos by Terry Henkel and Todd Elliott **c** original watercolor of the holotype by Mme. Goossens-Fontana from Heinemann (1959), reproduced with the permission of Botanic Garden Meise, Belgium.

**Figure 5.**
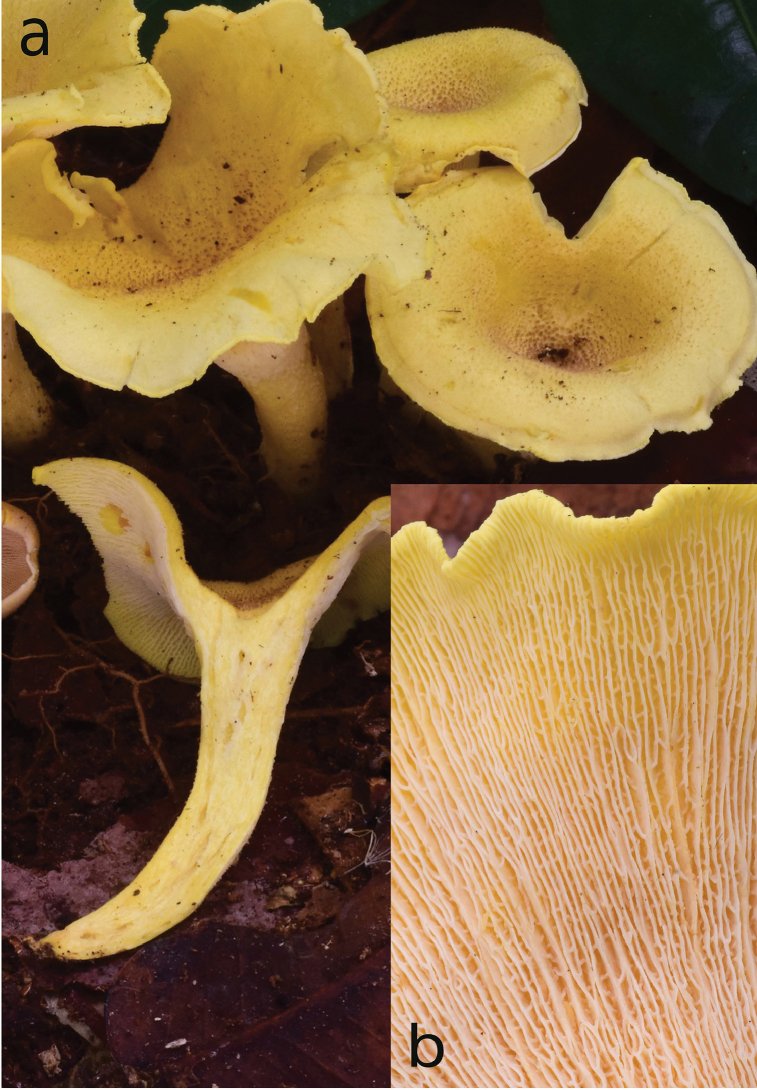
*Cantharellusluteopunctatus* (TH 10442). **a** Field habit of a collection that is more intensely yellow with distinctly yellowing context **b** detail of the hymenophore showing the crowded, thin and strongly anastomosing gill folds. Based on photos by Noah Siegel.

**Figure 6.**
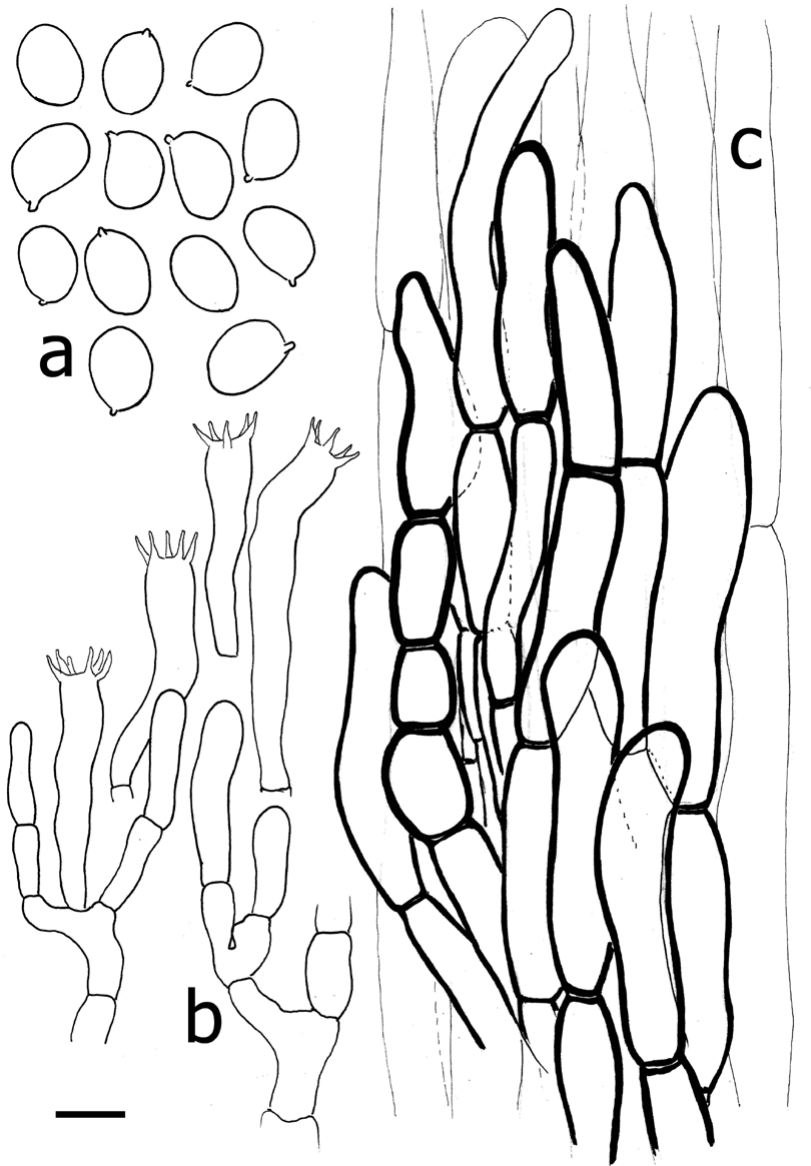
*Cantharellusluteopunctatus*. Microscopic features: **a** basidiospores **b** basidia and basidiola **c** detail of part of a squamula showing the terminal, thin- to slightly thick-walled hyphal extremities overlying the pileipellis. Scale bar: 10 µm but only 5 µm for basidiospores. Drawings: B. Buyck.

##### Description of the epitype.

*Basidiomata* scattered to occasionally caespitose. *Pileus* up to 65(–75) mm diam., initially broadly convex with shallow depression, extending outward and upward with age, becoming increasingly infundibuliform with downturned margin, deep golden yellow (2–3A4), beset with minute, conical erect tufts, these flesh-brown, more concentrated over the disc but extending and gradually more widely dispersed toward margin; intervening surface shiny-glabrous; margin irregularly crenulate, slightly wavy. *Hymenophore* composed of very thin, crowded, ridge-like gill folds, creamish to nearly concolorous with the pileus surface (2–3A3), occasionally developing tannish overtones with age (3–4A3), decurrent and fairly abruptly demarcated from the sterile stipe surface, discoloring slowly darker yellow to orangish where injured, repeatedly forking, also abundantly cross-connected between stipe and pileus margin at almost right angles, becoming increasingly tortuous and anastomosed with advanced age; edges even and concolorous. *Stipe* subequal or slightly tapering toward the base, (17–)24–43 × 4–8(–10) mm, concolorous with pileus, beset apically with conical, flesh-brown, erect tufts, longitudinally striate below; extreme base often developing some white mycelial tissue at the soil interface. *Context* solid, yellow, unchanging to increasingly yellow. *Odor* mildly chanterelle-like. *Taste* mild, nutty, pleasant, somewhat tardily acrid in young specimens. *Spore print* not obtained.

*Basidiospores* short-ellipsoid to ellipsoid, (5.4–)5.7–6.04–6.4(–7.1) × (3.9–)4.0–4.29–4.6(–5.0) µm, Q = (1.24)1.29–1.41–1.53(–1.79), smooth. *Basidia* quite short, mostly 30–40(–50) × 6–7 µm, (5–)6-spored. *Cystidia* none. *Subhymenium* cells mostly hardly wider than the basidium base, but locally more inflated parts make it somewhat intermediate between distinctly pseudoparenchymatous and filamentous. *Pileipellis* composed periclinal thin-walled hyphae of variable diameter, but most ca. 10 µm wide, that locally emit anticlinal tufts of short-septate chains of more or less inflated cells, with the largest cells in these chains distinctly zebroid incrusted and the more terminal cells distinctly thick-walled (up to 1 µm thick); terminal cells 30–60 µm long, mostly (6–)10–15 µm wide, subcylindric or clavulate to lageniform, with obtusely rounded to attenuated tips, never remarkably undulate or irregular in outline. *Clamp connections* absent.

CAMEROON. East Region: Dja Biosphere Reserve, Northwest Sector near the village of Somalomo, Upper Dja River Basin, within 2 km radius of Dja base camp located at 3°21'29.8"N, 12°43'46.9"E, 650 m a.s.l., 2 km SW of Dja base camp, under *Gilbertiodendrondewevrei*, coll. T. Henkel, 22 Nov 2016, TH 10285 (YA, **epitypus hic designatus**, duplicates at HSC G1264 and PC). MycoBank MBT 384670.

##### Additional specimens examined.

CAMEROON. East Region: Dja Biosphere Reserve, Northwest Sector near the village of Somalomo, Upper Dja River Basin, 1.4 km SW of Dja base camp, under *G.dewevrei*, coll. T. Henkel, 2 Sep 2014, TH 9921 (YA, HSC G1265, PC); 2 km SW of Dja base camp, under *G.dewevrei*, coll. T. Henkel, 29 Aug 2017, TH 10442 (YA, HSC G1266, PC).

##### Discussion.

*Cantharellusluteopunctatus* has long been considered as “uncomfortably close” to *C.densifolius*. Eyssartier (2001) found no significant microscopic differences between their holotypes, and suggested that *C.luteopunctatus* was likely a more yellowish color form of the latter species. For several decades following its original description *C.luteopunctatus* was not discussed in the literature, until recently by Eyi N’dong et al. (2011) who accepted it as an independent species. Both *C.luteopunctatus* and *C.densifolius* were also maintained as independent entities in a recent identification key to all African chanterelles (De Kesel et al. 2016). Our choice of epitype has been based on the specimen with the highest degree of similarity with the original macro- and microscopic description of *C.luteopunctatus*, and the original watercolor showing a species with a similar stature, color, and laterally compressed stipe (Fig. 4c).

Close reading reveals some differences between the original descriptions for *C.luteopunctatus* and *C.densifolius*. Apart from the difference in pileus color, the second most important difference, also noted by Eyi N’dong et al. (2011), concerns the configuration of the hymenophore. Gill folds were originally described for *C.luteopunctatus* as “irregularly forking and with many interstitial anastomosing veins”, versus those of *C.densifolius* which were “1–4 times forking, not interveined” (Heinemann 1958). Although the presence and frequency of anastomoses between gill folds can be highly variable among and within *Cantharellus* species, it remains nevertheless a very informative feature to characterize those species that appear always to be on one side of the continuum (De Kesel et al. 2016). In this particular case, the macromorphologies of recent collections suggest that this feature is consistent across, and different between, specimens of *C.luteopunctatus* and *C.densifolius*.

Our collections also demonstrate a difference in hymenophore color between the species, something that is not evident from Heinemann’s descriptions. Heinemann described *C.luteopunctatus* as having a yellow hymenophore, but does not indicate the color for the hymenophore of *C.densifolius*, although the original watercolor clearly shows it to be more or less ochraceous (see Fig. 2c here, and Heinemann 1959, Plate XXVI, fig. 6). Our collections confirm this ochraceous to dirty isabelline color of the hymenophore of *C.densifolius*, even when still relatively young, whereas the hymenophore color is more variable in *C.luteopunctatus* due to the translucent context above. The hymenophore of *C.luteopunctatus* is pure white when young, but it may also have pinkish tinges when the pileus surface is still densely covered by pinkish brown squamae, and then becomes more yellowish (which is the color mentioned in the original description) with maturation due to the yellowing context and absence of squamae over the expanded pileus margin. As in *C.densifolius*, the yellowing can be of variable intensity; for instance, the exposed context of TH 10442 is more strongly chrome yellow than that of the epitype (Fig. 5a).

Other considerable differences between *C.luteopunctatus* and *C.densifolius* include the surface structures of the pileus and stipe. In *C.luteopunctatus*, distinct central pileal squamae are erect, flesh brown to pinkish brown, and strongly separated and paler toward the margin. The pinkish color of the squamae was also mentioned in the original description of Heinemann (1958). In contrast, the pileus surface of *C.densifolius* is a continuous tomentum that lacks a pinkish flesh color and is woolly-fibrous, before breaking up concentrically in appressed fragments. Furthermore, in *C.luteopunctatus* the upper stipe surface has the same squamae as the pileus center, whereas in *C.densifolius* the stipe surface is smooth (compare Figs 2, 4, 5).

Micromorphologically, the basidiospores are nearly identical in both species (Table 1), but the pileipellis differs dramatically. In *C.luteopunctatus* the pileipellis is composed of fascicles of thin- to slightly thick-walled hyphae (corresponding to the erect squamae) that are recognizable on the background of more or less parallel, thin-walled hyphae of the interstitial surface, whereas in *C.densifolius*, the thicker-walled hyphae are not organized in fascicles. Moreover, in the latter species the distal cells of these thick-walled hyphae are much more undulate-sinuous in outline and narrower than those of *C.luteopunctatus*.

A final remark concerns the edibility of these Central African chanterelles: In Cameroon *C.luteopunctatus* basidiomata are mild-flavored and consumed by the indigenous Baka, while *C.densifolius* slowly develops a very strong bitterness and is not consumed by the Baka (T. Henkel, pers. obs.). While the bitter taste was also noted in the original description (Heinemann 1958), the first author did not detect bitterness for *C.densifolius* specimens from the Central African Republic.

#### Cantharellus tomentosoides

Taxon classificationFungiScleractiniaFungiidae

Buyck & V. Hofst. sp. nov.

828890

Figs 7 , 8

##### Diagnosis.

*Cantharellustomentosoides* is similar to *C.densifolius* in its low, blunt and crowded gill folds, overall yellowish brown color, identical basidiospores, and same habitat, but differs in its mostly smaller basidioma size, pileus surface texture, slightly more yellowish olive hymenophore color, and less thick-walled, less sinuous pileipellis extremities.

##### Gene sequences ex-holotype.

MG450685 (*tef*-1).

##### Etymology.

In reference to the species’ resemblance to its woodland sister-species, *C.tomentosus*.

##### Holotype.

CENTRAL AFRICAN REPUBLIC. Dzanga-Sangha Forest Reserve, near Bayanga, close to Bai-Hakou base camp, 02.859934N, 16.467492E, in monospecific *Gilbertiodendrondewevrei* forest, on bare sandy soil along trail at the entrance of the camp, 14 May 2016, Buyck 16.007 (PC0142485). MycoBank MBT 828890.

##### Description.

*Basidiomata* in small clusters, up to 40 mm high. *Pileus* 20–30 mm diam., thin and leathery, wavy with inrolled margin, young entirely hirsute-rugose, remaining lacerate-fibrillose to cottony in the center, elsewhere slightly rugose but lacking well-defined appressed scales, overall pale grayish brown with dark brown center, very early on becoming narrowly but strongly depressed centrally. *Stipe* slender, 6 mm diam., 20–30 mm high, rapidly elongating while pileus is still small, paler to concolorous with pileus margin, occasionally white at base; interior distinctly fistulose. *Hymenophore* composed of very crowded (up to 40/cm), low but comparatively thick and blunt gill folds, these 1 mm high, repeatedly forking, frequently fissuring over their full height, yellow with brownish tint, transitioning to warm egg-yolk yellow near extreme margin. *Context* leathery, whitish in the pileus, almost concolorous with the stipe surface, yellowing slowly. *Odor* agreeable, typical. *Taste* mild. Spore print not obtained.

*Basidiospores* short-ellipsoid to ellipsoid, (5.8–)6.0–6.36–6.7(–7.1) × (3.9–)4.0–4.27–4.5(–5.0) µm, Q = (1.3–)1.4–1.49–1.6(–1.7), smooth, hyaline. *Basidia* short and narrow, 30–38 × 6–8 µm, mostly five-spored. *Subhymenium* pseudoparenchymatous, composed of short, barely inflated cells that are slightly wider than the basidium base. *Cystidia* none. *Pileipellis* composed of almost thin-walled to slightly refringent hyphal extremities, mostly 4–8 µm wide; terminal cells rather short, mostly 20–40(–50) µm long, subcylindrical, regular in outline, broadly rounded at the apex; walls refringent, not thickened. *Clamp connections* absent.

**Figure 7.**
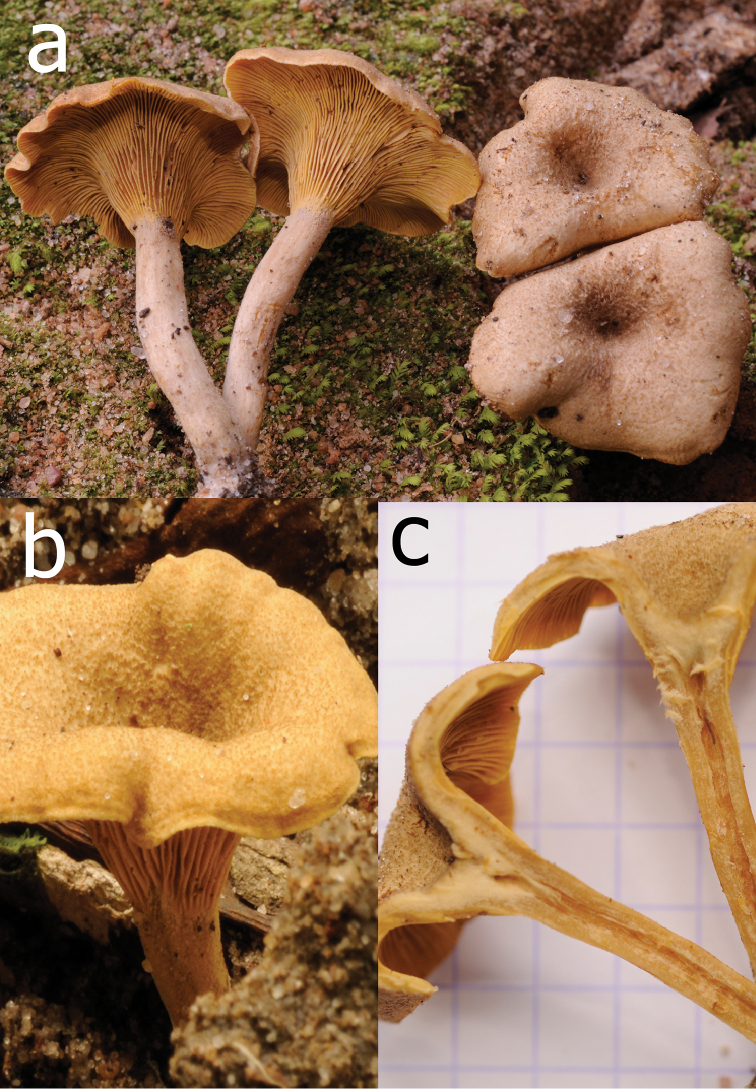
*Cantharellustomentosoides* (holotypus, Buyck 16.007). **a** Field habit **b** detail of the pileus surface **c** Longitudinal section showing the fistulose stipe. Photos: B. Buyck.

**Figure 8.**
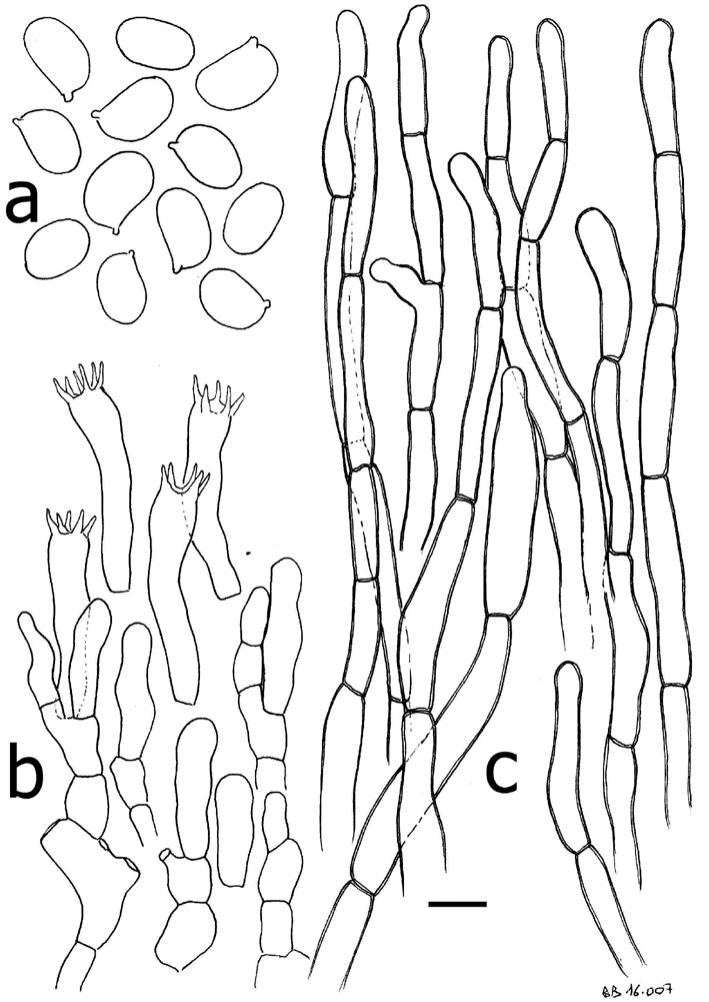
*Cantharellustomentosoides*. Microscopic features: **a** basidiospores **b** basidia and basidiola **c** densely septate and nearly thin-walled hyphal extremities of the pileipellis. Scale bar: 10 µm but only 5 µm for basidiospores. Drawings: B. Buyck.

##### Discussion.

*Cantharellustomentosoides* is a rain forest species that is phylogenetically sister to *C.tomentosus* Eyssart. & Buyck (Fig. 1), for which it was initially mistaken in the field. The latter species was described from miombo woodland in Tanzania and was documented from Burundi by Buyck (1994) under the local name ‘nyarumpu’. Apart from its different habitat, *C.tomentosus* differs from *C.tomentosoides* in its slightly narrower basidiospores (6–6.98–8 × 3.5–3.92–4.5 μm, Q = 1.5–1.79–2.1), narrower hyphal extremities of the pileipellis, more brownish gill folds, and its nearly smooth to faintly squamose pileus surface (Buyck et al. 2000).

*Cantharellustomentosoides* resembles *C.densifolius* in its similarly crowded gill folds, overall yellowish brown coloration and identical basidiospores, but differs in its mostly smaller size, different texture of pileus surface, slightly different color of hymenophore, and thinner-walled hyphal extremities at the pileus surface. Additionally, these two species are phylogenetically distinct (Fig. 1).

#### Cantharellus densilamellatus

Taxon classificationFungiScleractiniaFungiidae

Buyck & V. Hofst. sp. nov.

828893

Figs 9 , 10

##### Diagnosis.

*Cantharellusdensilamellatus* resembles *C.densifolius* in its overall yellowish to orange-brown color, but differs in its thinner and comparatively well-developed gill folds with less blunt edges, smaller size, nearly thin-walled, less undulate hyphal extremities at the pileus surface, more elongate basidiospores, and its seasonal woodland habitat.

##### Gene sequences ex-holotype.

JX193014 (published in Buyck et al. 2014).

##### Etymology.

“densilamellatus”; referring to the relatively close spacing of the gill folds.

##### Holotype.

TANZANIA. Msanga village, in very degraded woodland with *Brachystegia*, 24 April 1998, Buyck 98.013 (PC0084126). MycoBank MBT 828893.

##### Description.

*Basidiomata* small to medium-sized. *Pileus* up to 60 mm diam., first centrally depressed and with a downturned margin, then becoming more depressed as the margin spreads out, fleshy in the center, but increasingly thin fleshed toward the margin and there often striate or radially splitting; margin regular to slightly wavy-lobed; surface dry, with a pale to dark brown to reddish brown tomentum (5DEF6–8) contiguous over disc, toward margin tomentum separating concentrically into a pale yellowish cream (3A2–3) areolate pattern. *Hymenophore* composed of thin, well-developed gill folds, 2–3 mm high, densely spaced (> 10/cm) but not crowded, forking, not anastomosing, often splitting transversely through their entire height, uniformly pale yellow (3A4), brighter than the pileus margin and stipe. *Stipe* central, up to 40 mm long, 6–11 mm wide, subcylindrical to slightly wider near the base, rapidly elongating before the pileus margin starts to spread, concolorous with the pileus margin, distinctly finely squamulose over apical portion, compact in section. Context off-white to pale cream, weakly yellowing. *Odor* fruity. *Taste* mild. *Spore print* off-white to very pale yellowish.

*Basidiospores* narrowly ellipsoid to almost elongate, often reniform or peanut shaped, 6.7–7.05–7.4(–7.9) × (3.3–)3.4–3.65–3.9(–4.0) µm, Q = (1.7–)1.8–1.94–2.1(–2.3), smooth. *Basidia* rather short, 35–50(–58) × 6–7 µm, (4–)5–6-spored; basidioles mostly clavate. *Cystidia* none. *Subhymenium* pseudoparenchymatous, composed of irregular, slightly inflated cells. *Pileipellis* a cutis of interwoven hyphal extremities forming slender chains of subcylindrical cells, these quite regular in outline, with thin to very slightly thickened and refringent walls; terminal cells (25–)30–45(–65) × (3–)4–7 µm, subcylindrical or sometimes very slightly inflated in the lower or middle portion, obtusely rounded at the tip or slightly constricted subapically. *Clamp connections* absent.

**Figure 9.**
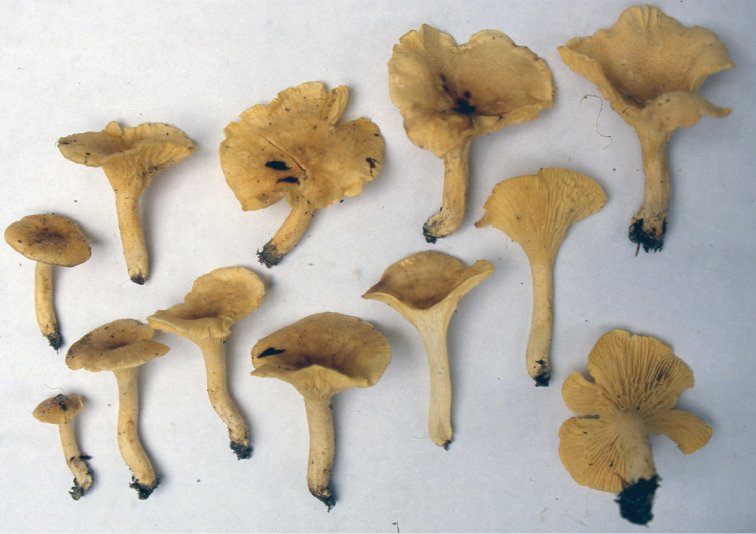
*Cantharellusdensilamellatus* (holotypus, Buyck 98.013). Aspect of fresh specimens. Photo: B. Buyck.

**Figure 10.**
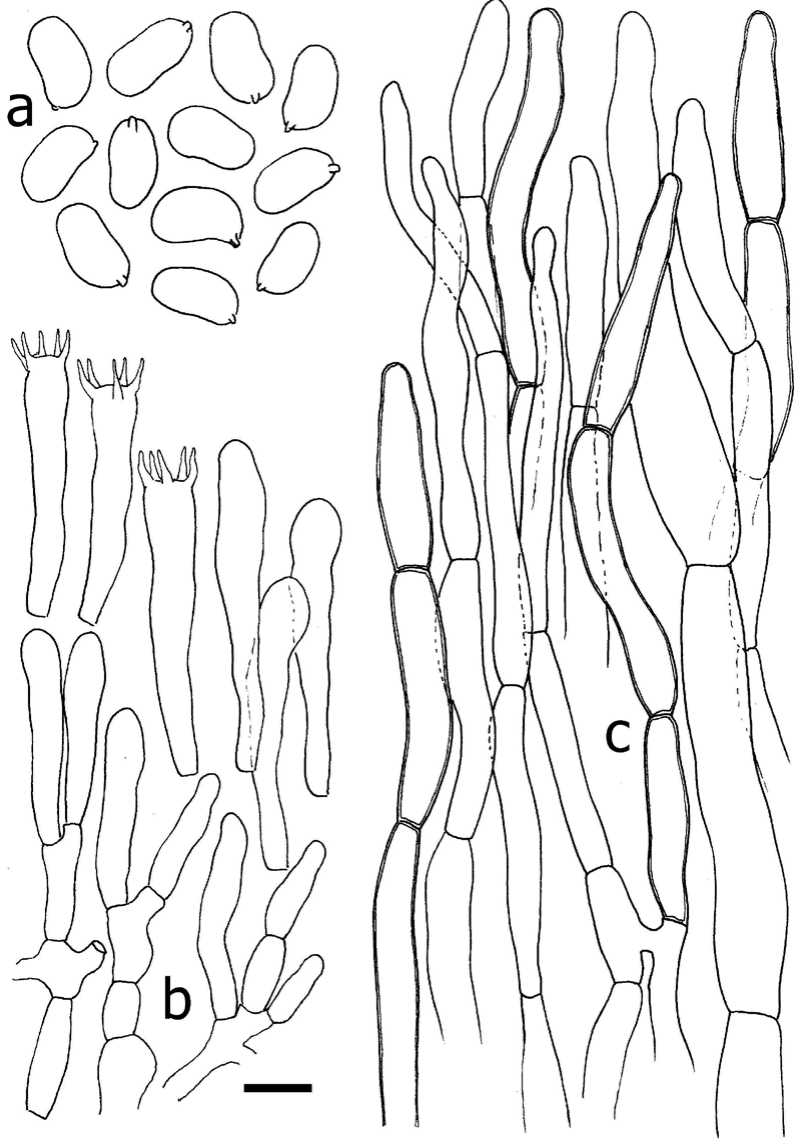
*Cantharellusdensilamellatus* (holotypus, Buyck 98.013). Microscopic features: **a** basidiospores **b** basidia and basidiola **c** thin-walled to slightly refringent hyphal extremities of the pileipellis. Scale bar: 10 µm but only 5 µm for basidiospores. Drawings: B. Buyck.

##### Discussion.

*Cantharellusdensifolius* has long been the only available name for yellowish brown, clampless chanterelles in Africa with a squamulose pileus and crowded gill folds. Indeed, the holotype collection of *C.densilamellatus* reported here was initially identified as *C.densifolius* in Buyck et al. (2000) and, in the absence of any reliable concept for Heinemann’s species, was even maintained as *C.densifolius* in the multigene *Cantharellus* phylogeny of Buyck et al. (2014). Although more than one woodland species might have been referred to as ‘*C.densifolius*’, *C.densilamellatus* as described here is undoubtedly one of the more common and widespread inhabitants of the Zambezian miombo woodlands. It differs from the true *C.densifolius* not only in its habitat preference, but also in its more elongated basidiospores, which are very similar to those of *C.tomentosus* Eyssart. & Buyck (another, but much less common, woodland species with crowded gills and much darker pileus surface and hymenophore – see Buyck 1994 [as ‘nyarympu’, its Kirundi vernacular name] and Buyck et al. 2000). *Cantharellusdensilamellatus* further differs micromorphologically from *C.densifolius* in its more regular, less undulate and thinner-walled hyphal extremities of the pileus surface (Fig. 10). While the phylogenetic analysis presented here (Fig. 1) shows a close relationship of *C.densilamellatus* with *C.luteopunctatus*, the latter species differs in its pinkish, erect squamae, bright yellow pileus color, and initially white and strongly anastomosing gill folds.

## Conclusion

Phylogenetic analysis including the newly obtained sequence data demonstrated that *C.densifolius* and *C.luteopunctatus*, here epityped, belong in the same subgenus but in different subclades. Additionally, the name *C.densifolius* has been consistently misapplied to at least one, and possibly several, similar taxa from the African woodland area (De Kesel pers. comm., Buyck 1994, Buyck et al. 2000, Härkönen et al. 2015). One of these woodland species described here, *C.densilamellatus*, is very different morphologically from, but phylogenetically sister to, *C.luteopunctatus*. *Cantharellustomentosoides* is a new species that is morphologically similar to, but phylogenetically distinct from, *C.densifolius* and is from the same local habitat in the *G.dewevrei* rain forest. These results, along with the continuing discovery of new, morphologically unique African chanterelles, emphasize the importance of Africa as a global diversity hotspot for *Cantharellus* (Buyck 2016, De Kesel et al. 2016, Buyck et al. 2017).

## Supplementary Material

XML Treatment for Cantharellus densifolius

XML Treatment for Cantharellus luteopunctatus

XML Treatment for Cantharellus tomentosoides

XML Treatment for Cantharellus densilamellatus
